# Single-cell genomics links targeted functional manipulations to efficacy-associated chromatin signatures in CAR T cells

**DOI:** 10.21203/rs.3.rs-9859689/v1

**Published:** 2026-06-29

**Authors:** Kole R. DeGolier, Stephanie DeVoe, Marwan. A. Abushawish, Angela Prete, Terry J. Fry, James P. Scott-Browne

**Affiliations:** 1 Department of Immunology and Genomic Medicine, National Jewish Health, Denver, CO, USA, 80206; 2 Immunology Graduate Program, University of Colorado Anschutz Medical Campus, Aurora, CO, USA, 80045; 3 Department of Pediatrics, University of Colorado Anschutz Medical Campus, Aurora, CO, USA, 80045; 4 Department of Immunology & Microbiology, University of Colorado Anschutz Medical Campus, Aurora, CO, USA, 80045

**Keywords:** Leukemia, CAR T cells, tonic signaling, affinity, barcoding, chromatin, ATAC-seq

## Abstract

Chimeric antigen receptor (CAR) T cells produce extraordinary remission rates in some hematologic tumors, results inconsistently replicated across malignancies and/or target antigens. Anti-tumor efficacy can be enhanced by modifying CAR architecture or T cell differentiation state. Pooled screening methods to identify effects of modifications are often restricted to *in vitro* readouts of abundance or transcriptome, limiting ability to interrogate biology and project long-term T cell fates. We use genetically-encoded barcodes to track the effects of diverse functional manipulations on pooled murine and human CAR T cell chromatin states using scATAC-seq. We report stable and transient transcription factor activities programmed by cytokine concentration during *in vitro* expansion and altered *in vivo* effector differentiation programs driven by modifications to CAR antigen binding domain construction. These data establish genetic barcoding to tie targeted functional manipulations to single CAR T cell chromatin profiles *in vitro* and *in vivo*, providing insights toward augmented therapeutic efficacy.

Cancer immunotherapy via adoptive transfer of chimeric antigen receptor (CAR) T cells can mediate remarkable patient outcomes^1–4^. Nonetheless, heterogeneity between autologous products and within each T cell pool drives variability in clinical responses^5–7^. Modifying CAR structure or T cell manufacturing conditions improves therapeutic efficacy in preclinical studies and clinical trials^8–16^, but tying pre-transfer manipulations to long-term T cell fates which contribute to patient outcomes has been challenging. We and others have shown that chromatin accessibility reflects activity of transcription factors (TFs) controlling gene expression programs driving T cell development, activation, and function^17–20^. Conversely, the transcriptome provides a snapshot of cellular status. Single-cell chromatin accessibility assays elucidate heterogeneity in cell fates via projection of genome-wide TF activity and assessment of linked regulatory elements to determine how transcriptional programs may be controlled.

Spear-ATAC (single-cell perturbations with an accessibility read-out using assay for transposase accessible chromatin with sequencing) was previously used to determine the effects of CRISPR/Cas9 perturbations on chromatin profiles of pooled cell lines *in vitro* at single cell resolution^21^. Here, we extend Spear-ATAC, using genetically-encoded barcodes to track the chromatin signatures of primary murine and human CAR T cells cultured under different conditions or expressing CARs with different antigen binding domains. Pooled analysis increases throughput while reducing batch effects and use of sequencing reagents and research animals. Treating separate tumor-bearing hosts with T cells of differing potencies can produce disparate tumor burdens and T cell antigen exposure across groups, confounding comparisons. Thus, assaying pooled T cells *in vivo* also provides the distinct advantage of homogenous environment and tumor exposure. Finally, readout of barcode abundance facilitates a large-scale competition assay between T cells with distinct modifications.

Our data reveal stable and transient changes in TF activity dictated by interleukin-2 (IL-2) concentration during murine CAR T cell expansion. We also profile human CD22-CAR variants and report antigen-independent 4–1BB signaling, considered essential for clinical activity of CD22-CARs^2,13^, drives altered *in vivo* effector programs which may be detrimental to anti-tumor efficacy. Overall, we establish pooled fate tracking with scATAC-seq, using genetic barcodes linked to targeted functional manipulations, for decoding chromatin-level determinants of CAR T cell function *in vitro* and *in vivo*.

## RESULTS

### Species-associated barcoding guides optimal filtering parameters to balance fidelity and cell capture efficiency

**Figure 1:**
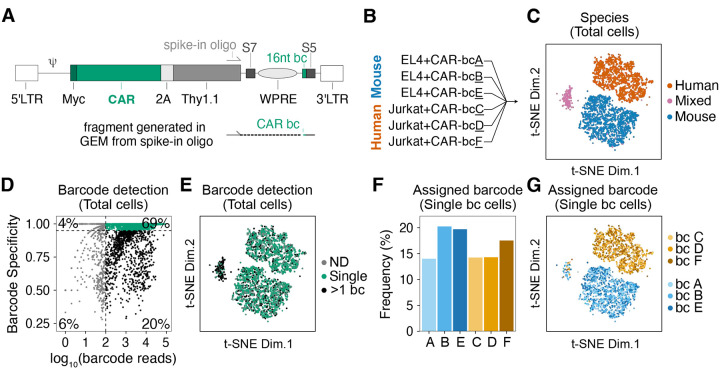
Species-associated barcoding to optimize *in silico* filtering parameters for genetically-barcoded scATAC-seq **1A:** Diagram of gammaretroviral murine CAR construct with barcode cassette. Constructs contain 5’UTR, Myc-tagged CAR transgene, P2A, murine Thy1.1 reporter, barcode cassette containing WPRE3 and 16bp DNA barcode flanked by Nextera sequencing adapters, and self-inactivating (SIN) 3’LTR. A spike-in oligo primes amplification of the barcode fragment during scATAC capture to facilitate later enrichment of barcode library. **1B:** Experimental schematic: Three distinctly-barcoded CAR constructs per species were transduced into either murine EL4 T cell lymphoma or human Jurkat T cell leukemia lines before pooling and capture for scATAC-seq. **1C:** t-Distributed Stochastic Neighbor Embedding (t-SNE) visualization of all pooled cells, colored by species. **1D:** Barcode detection in all pooled cells by scATAC-seq, with cell filtering thresholds indicated for both ‘barcode specificity’ (proportion of barcode reads matching barcode with most reads) and log_10_(# barcode reads per cell). Cells in top right quadrant (green) meet inclusion thresholds. **1E:** t-SNE visualization of cells that met QC cutoffs, colored by inclusion criteria (‘Single’ have been confidently assigned a barcode, ‘>1 bc’ are barcode multiplets, and ‘ND’ are not designated). **1F:** Frequency of cells meeting inclusion criteria (Single) which were assigned to each barcode. **1G:** t-SNE visualization of cells meeting inclusion criteria (Single), colored by barcode.

To generate a Spear-ATAC-compatible vector, we modified our human-CD19-reactive murine CAR construct^22^ to embed a DNA barcode flanked by Nextera sequencing adapters downstream of the mouse Thy1.1 (mThy1.1) reporter (Figure 1A). To test barcoding fidelity, we transduced murine EL4 and human Jurkat T cell lines with three distinct genetic barcodes each before sorting for mThy1.1+ cells using fluorescence-activated cell sorting (FACS) and pooling for scATAC-seq (Figure 1B, S1A). Barcode transduction did not alter expected scATAC-seq fragment sizes or standard QC metrics (Figure S1B–C). Barcodes were amplified by spike-in of a barcode-amplification oligonucleotide during capture, followed by nested PCR to enrich and index barcode-containing fragments from the final scATAC library (Figure S1D–E). We filtered for cells meeting QC thresholds before visualization by t-Distributed Stochastic Neighbor Embedding (t-SNE). These comparisons revealed distinct populations of human, mouse and doublet cells determined by the numbers of genomic reads in each cell mapping to mouse and human genomes (Figure 1C). We assigned barcode identity to each cell based on ‘barcode specificity’ (proportion of barcode reads matching barcode with most reads) and number of barcode reads for the barcode with most reads. We compared classification of cell species based on assigned barcode versus the determination based on numbers of reads mapping to each genome (Figure 1D). We found that increasing the specificity threshold reduced proportions of incorrect species assignments, while increasing the reads threshold reduced multiplets (Figure S1F). A sensitivity threshold of 0.95 with at least 100 reads maximized cell inclusion and minimized multiplets or incorrect species assignments across several barcode constructs (Figure 1D–E, S1F–G). Importantly, with equal pooling of distinctly barcoded cells we recovered similar proportions of each barcode, indicating barcode sequence did not affect recovery rate (Figure 1F–G). These analyses validate the approach and empirically determined *in silico* thresholds to ensure data quality and method fidelity for downstream experiments.

### Culture condition-associated barcoding of primary murine CAR T cells reveals stable and transient transcriptional programs dictated by IL-2 concentration

**Figure 2:**
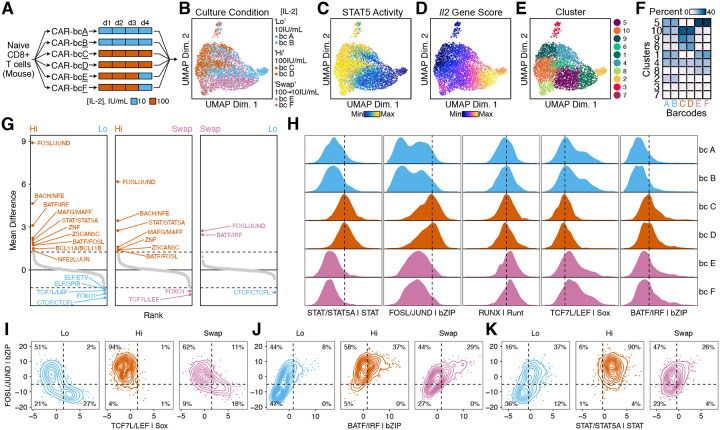
Culture condition-associated barcoding of primary murine CAR T cells reveals stable and transient transcriptional programs dictated by IL-2 concentration **2A:** Experimental schematic: Two distinctly barcoded CAR constructs per IL-2 condition (‘Lo,’ 10IU/mL; ‘Hi,’ 100IU/mL; ‘Swap,’ 100IU/mL changed to 10IU/mL 16 hours prior to harvest) were transduced into murine CD8 T cells before pooling and capture for scATAC-seq. **2B-E:** Uniform Manifold Approximation and Projection (UMAP) visualization of all pooled cells, colored by IL-2 condition **(B)**, ChromVAR-computed STAT5 motif-associated chromatin accessibility (z-score) **(C)**, *Il2* gene score **(D)**, or Seurat-computed cluster **(E)**. **2F:** Proportion of cells assigned each barcode per cluster (percentages computed by column). **2G:** Difference in mean (deviation) of ChromVAR z-scores across the three pairwise comparisons. **2H-K:** ChromVAR-computed motif-associated chromatin accessibility z-scores for all cells assigned each barcode, for the indicated transcription factor motifs **(H)**, or the indicated pairwise motif comparisons **(I-K)**.

The T cell growth factor IL-2 plays a central role in protocols for expanding tumor-specific T cells for adoptive cell therapy^23,24^. However, IL-2 concentrations during culture can vary, with few specific comparisons to characterize long-term effects on T cell differentiation^25^. To interrogate the effects of IL-2 concentration on murine CAR T cells, we used Spear-ATAC to compare chromatin accessibility of T cells cultured in three IL-2 supplementation conditions: 100IU/mL (‘Hi’), 10IU/mL (‘Lo’), or a ‘Swap’ condition which started at 100IU/mL before switching to 10IU/mL (Figure 2A). We transduced T cells with two distinctly barcoded CAR constructs for each culture condition (Hi/Lo/Swap) before sorting and pooling for scATAC-seq (Figure 2A, S2A). We anticipated that Swap could emulate IL-2 deprivation experienced by T cells post-transfer (a driver of reduced persistence) and reveal stable versus transient transcriptional programs^26^. After filtering and QC (Figure S2B–D), we observed distinct accessibility profiles for each condition, replicable across each set of two barcodes (Figure 2B, S2E–F). To infer global TF activity and gene expression, we computed ChromVAR^27^ z-scores and gene scores. T cells with high STAT5 motif-associated z-scores (Hi) versus high *Il2* gene scores (Lo and Swap) were discrete, consistent with exclusivity of populations sending or receiving IL-2 priming signals^28^ (Figure 2C–D). Unbiased clustering separated cells by culture condition, with overlap between Lo and Swap supporting plasticity of some IL-2-dependent programs (Figure 2E–F). Ranked pairwise comparisons revealed strong condition-dependent differences in TF activity (Figure 2G). STAT5 activity was amplified in Hi and some Lo cells, while low activity in Swap indicated uniform IL-2 dependance. FOSL/JUND activity was bimodal in Lo, with increased activity in Hi dropping after Swap. RUNX activity between Hi and Lo was similar, with increased activity in Swap. TCF/LEF activity dropped in Hi compared to Lo, recovering in Swap. Finally, BATF/IRF only minorly retracted upon Swap (Figure 2H). Globally ranked TF activity revealed FOSL/JUND as having the greatest variability between conditions by a large margin (Figure S2G). Plotting this motif in tandem with others revealed unique patterns of coregulation (BATF/IRF, STAT5) or opposition (TCF/LEF) (Figure 2I–K). Thus, IL-2 concentration during CAR T cell transduction and expansion has a prominent impact on TF activity, with heterogeneity in stable versus transient programs.

### *In vitro* and *ex vivo* coupling of CD22-CAR architecture-associated barcodes with chromatin accessibility in human T cells exposes temporal patterns associated with adoptive transfer

CD22-targeted CARs mediate high remission rates in B-lineage acute lymphoblastic leukemias (B-ALL) and lymphomas that are resistant to CD19-CAR therapy, but patients often relapse with low leukemic CD22 density (CD22^Lo^)^2^. Prior work demonstrates that transcriptional perturbations including overexpression of RUNX2, T-bet, or c-JUN can increase CAR functionality against antigen-low leukemias^17, 29, 30^ but programs necessary for robust responses to antigen-low tumors remain unclear.

**Figure 3:**
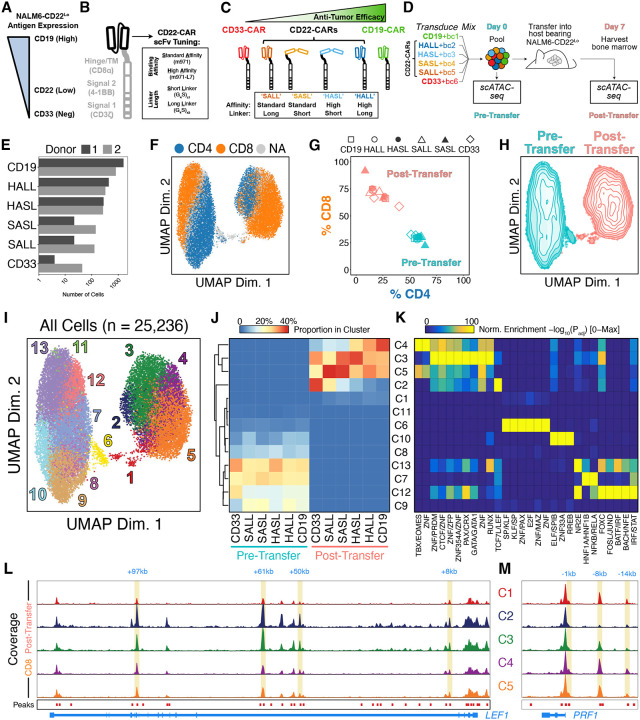
*In vitro and ex vivo* coupling of CD22-CAR architecture-associated barcodes with chromatin accessibility in human T cells exposes temporal and efficacy-linked chromatin accessibility signatures **3A-C:** Schematics depicting: Relative antigen expression on NALM6-CD22^Lo^ B-lineage acute lymphoblastic leukemia. **(A)**. Conserved CAR architecture and modifications to scFv binding affinity or tonic-signaling profile (linker length) for CD22-CARs. **(B)**. Previously observed efficacy against NALM6-CD22^Lo^ for CD22-CARs, benchmarked against the CD19-CAR (positive control) and CD33-CAR (negative control) **(C)**. **3D:** Experimental schematic: Distinctly barcoded CARs with the architectures described in 3C were transduced into primary human T cells and pooled at equal ratios for scATAC-seq profiling (‘Pre-Transfer) or adoptively transferred (1e6 CAR+ cells/mouse) into mice which had received 1e6 NALM6-CD22^Lo^ 3 days prior. 7 days after CAR transfer, bone marrow was harvested and pooled for scATAC-seq profiling (‘Post-Transfer’). Data in **3E-3K** are from pooled T cells from both donors and both timepoints (n = 25,236 cells from n = 2 biological donor replicates, profiled pre- and post-transfer) **3E:** T cell abundance for each CAR in the scATAC-seq pool, separated by donor. **3F:** Uniform Manifold Approximation and Projection (UMAP) visualization of coreceptor assignment (CD4, CD8 or not assigned (NA)) **3G:** Proportion of cells with each coreceptor assignment, stratified by CAR barcode assignment and timepoint. **3H-I:** UMAP colored by timepoint **(H)** or Seurat-computed cluster **(I)**. **3J:** Proportion of cells assigned each barcode across clusters (percentages computed by column). **3K:** Normalized enrichment by column (−log10(P_adj_)) for motif-associated chromatin accessibility (z-scores) within differentially accessible peaks for each cluster, for top 6 motifs which were differentially accessible within each cluster (can be shared across clusters). **3L-M:** Genomic chromatin accessibility tracks surrounding the indicated gene loci for CD8 CAR T cells, pseudobulked and stratified by cluster. Boxes indicate peaks which were differentially accessible between CD8 CAR T cells within Clusters 3 and 4 at putative or established regulatory elements surrounding each gene locus. Distance from TSS indicated above.

We previously modified the NALM6 B-ALL cell line to express low levels of CD22 (NALM6-CD22^Lo^)^2^ and verified high endogenous CD19 expression and absence of CD33 (Figure 3A, S3A). We also previously demonstrated that CD22-CAR antigen binding domain architecture determines graded anti-tumor efficacies of CD22-CAR T cells against NALM6-CD22^Lo^ with the greatest activity observed using a high affinity single chain fragment variable (scFv) with a long linker between heavy and light chain, reducing tonic signaling^9^. CD22-CARs tested incorporated standard (SA) or high affinity (HA) binders with short (SL) or long linkers (LL), totaling four CD22-CAR variants (Figure 3B, C). We confirmed expression of each barcoded CAR variant on T cells (Figure S3B–D).

To understand how CD22-CAR affinity and tonic signaling modulate T cell fate, we employed Spear-ATAC to track pooled T cells expressing different CD22-CARs during response to NALM6-CD22^Lo^, including internal positive (CD19-CAR) and negative (CD33-CAR) controls (Figure 3C–D, S3E–J). Relative CD22-CAR abundance was replicable across donors and in accordance with previously reported anti-tumor efficacies of the CAR variants^9^ (Figure 3E). We combined two donors and two timepoints (n = 25,236 total cells) to perform global chromatin accessibility analyses. We assigned CD4 or CD8 coreceptor subset labels based on cutoffs for *CD4*, *CD8A* and *CD8B* gene scores (Figure 3F). All CARs had a greater proportion of CD4 T cells after transduction (‘Pre-Transfer’), while transfer into leukemia-bearing hosts (‘Post-Transfer’) resulted in accumulation of CD8 cells, regardless of target antigen density^9^ (Figure 3G, S4A–B). Uniform Manifold Approximation and Projection (UMAP) analysis revealed firm separation by timepoint, even with the CD33-CAR, indicating that distinct environmental cues between *in vitro* culture and *in vivo* xenograft drive critical antigen-independent programs in adoptively transferred T cells (Figure 3H, S4C).

### Interspecies mapping of IL-2-dependent murine genomic regions to human T cells supports decreased IL-2 availability as a key determinant of altered chromatin accessibility upon xenogeneic transfer

The distinctions between Pre-Transfer and Post-Transfer in all CARs (Figure 3G–H, S4A–C) suggests adoptive transfer as a major determinant of chromatin accessibility, independent of antigen exposure. We hypothesized that reduced IL-2 concentration experienced by T cells upon transfer contributed to distinct chromatin profiles. Cells with the highest STAT5 activity (Pre-Transfer, CD8), were distinct from those with the highest *IL2* gene scores (Post-Transfer, CD4) (Figure S4D–E). To further interrogate this, we created reference sets of human peaks with high sequence similarity to regions of the mouse genome that were differentially accessible between Hi and Lo IL-2 conditions. We scored our human T cell data on the reference peaks which were more accessible in Hi than Lo (‘Hi > Lo’) or vice versa (‘Hi < Lo’). Pre-Transfer cells mirrored the STAT5 activity data, with the CD8 compartment showing the highest average z-score for Hi > Lo peaks, indicative of IL-2 signaling. Conversely, Post-Transfer cells mirrored *IL2* gene scores, exhibiting high z-scores for peaks enriched in Hi < Lo. Slightly lower z-scores in CD4 cells were consistent with a role in IL-2 production (Figure S4F–I). The central role of IL-2 was also apparent from decreased accessibility across the *IL2RA* locus after transfer (Figure S4J). Together, these data pinpoint the drop in IL-2 concentration upon adoptive transfer as a key determinant of chromatin accessibility and support a helper role for CD4 CAR T cells, producing IL-2 *in vivo*.

### Efficacy-associated chromatin signatures revealed by global profiling of human T cells

To define global distinctions in chromatin signatures between CD22-CAR architectures that could be linked to anti-tumor efficacy, we clustered cells from both donors at both timepoints (Figure 3I). Strong efficacy-delineated CAR enrichment patterns were observed within the Post-Transfer timepoint (Cluster 2–5) (Figure 3J). HALL shared enrichment patterns with the strongly responding CD19-CAR (Cluster 4), and SALL clustered with the bystander CD33-CAR and SASL (Clusters 2,5). The two SL CD22-CARs coincided across timepoints, signifying linker-driven programming (Cluster 3,7 in Figure 3I–J). We also observed shared enrichment of SL CARs in CD4-associated Cluster 3, and CD19 and HALL in CD8-associated Cluster 4 (Figure 3J). We next identified differentially accessible peaks in each cluster compared to all cells and compared TF motif enrichment within these groups of regions to infer TF activities. Peaks linked to Cluster 4 were enriched for a T-box motif and a zinc-finger (ZNF) motif with a similar sequence, indicative of robust effector differentiation. In contrast, peaks linked to the CD33 and SALL CAR-enriched Cluster 2 were associated with the TCF7L/LEF motif, suggesting a resting or memory-like population. Finally, ZNF, GATA, RUNX and NFκB motifs were enriched in the SL CAR-associated Cluster 3, indicating restricted or altered effector differentiation programs (Figure 3K). Limited CD8 effector programming in SL CAR-enriched Cluster 3 was validated by patterns of increased (*LEF1)* or decreased (*PRF1*) chromatin accessibility relative to Cluster 4 (Figure 3L–M). In summary, high anti-tumor potential is linked to strong CD8 effector programming seen in CD19 and HALL CARs, and lower anti-tumor potential with altered effector programs (SL CARs) or resting/memory-like programs (SALL/CD33-CARs).

### CD22-CAR architectures drive distinct *in vivo* effector transcriptional programs in CD4 and CD8 CAR T cells

**Figure 4:**
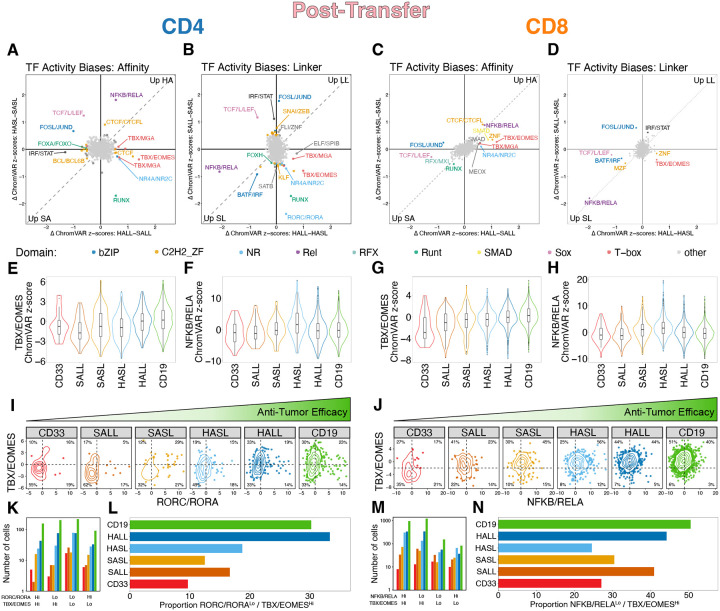
CD22-CAR architectures drive distinct *in vivo* NFκB and effector-associated transcriptional programs during the anti-leukemia response Data on left side of page (panels A, B, E, F, I, K, L, O, P) are from CD4 CAR T cells and data on right side of page (panels C, D, G, H, J, M, N, Q, R) are from CD8 CAR T cells. **4A-D:** CD22-CAR architectural biases in transcriptional activity for transcription factors binding the indicated motifs, driven by either CD22-CAR affinity **(A, C)** or linker **(B, D)**. Coordinates are calculated as the difference between ChromVAR motif-associated chromatin accessibility z-scores for the comparisons as indicated on each axis. Points closer to the y = x diagonal line are more directly biased by affinity or linker as indicated, while points away from the diagonal exhibit preferential bias by either the x-axis or y-axis comparison. Cutoff value of >0.5 in any direction was applied. **4E-H:** Comparisons of ChromVAR motif-associated chromatin accessibility z-scores for all cells assigned each barcode for the indicated motifs. **4I-J**: Pairwise comparisons of ChromVAR motif-associated chromatin accessibility z-scores for all cells assigned each barcode for the indicated sets of motifs. **4K:** Quantifications of total cell numbers in each quadrant, stratified by CAR. **4L:** Proportions of each CAR in the indicated quadrant. **4M:** Quantifications of total cell numbers in each quadrant, stratified by CAR. **4N:** Proportions of each CAR in the indicated quadrant.

To further elucidate transcriptional programs driven by differences in CD22-CAR architecture, we separated CD4 and CD8 CAR T cells and plotted differences in mean ChromVAR z-scores between CD22-CARs after grouping by affinity or linker. Activity of several TF families showed affinity (NFκB, T-box) or linker (NFκB, MZF, BATF/IRF) biases. We also observed CAR-specific TF activity preferences with HALL (T-box), HASL (Sox), SALL (IRF/STAT), and SASL (RORC, RUNX) (Figure 4A–D). Interestingly, we also found the magnitude of linker-driven differences was greater in CD8 than CD4 T cells Pre-Transfer, with the opposite pattern Post-Transfer, indicating tonic 4–1BB signal may robustly program CD4 T cells after transfer, while pre-programming CD8 T cells (Figure 4A–D, S5A–D). We observed several consistent and robust differences in TF activity in CD4 and CD8 T cells, with T-box activity associated with efficacy, NFκB activity showing a SL bias and nuclear receptor (NR, likely Rorγt) activity showing SASL bias in CD4 T cells, highlighting the substantial influence of CD22-CAR architecture on effector and helper-lineage differentiation (Figure 4A–H, S5A–L). To interrogate potential connections between TF activities, we made pairwise comparisons stratified by CAR and coreceptor assignment (Figure 4I–N). The SASL architecture drove a high proportion of CD4 cells with NR activity and non-canonical T-box/NR double positive cells (Figure 4I,K,L). T-box activity showed an efficacy-associated gradient, highest in HALL and CD19 CARs for both CD4 and CD8 subsets (Figure 4E,G,I,J). Finally, we observed that SL CD22-CARs blunted proportions of RORC/RORA^Lo^/TBX/EOMES^Hi^ (CD4) and NFKB/RELA^Lo^/TBX/EOMES^Hi^ or TCF7L/LEF^Lo^/TBX/EOMES^Hi^ (CD8) T cells, populations highly enriched in both efficacious HALL and CD19 CARs (Figure 4L,N, S5H,K,L). Together, these data demonstrate that CD22-CAR architectures drive distinct NFκB, T-box, NR and TCF/LEF activities, with SL CARs promoting altered helper-lineage and effector transcriptional programs in CD4 and CD8 CAR T cells.

## DISCUSSION

Optimization of methodologies to augment anti-tumor T cell responses is essential for successful cellular immunotherapies. Targeted functional manipulations can include adjustment of culture conditions (e.g. by modifying cytokine milieu or addition of small molecules), modifications to synthetic receptors to enhance antigen binding properties, structural regions, or signaling domains, or genetic manipulations (e.g. CRISPR/Cas-based perturbations)^8–16^. Pooled screens allow for high throughput profiling in a homogenous environment and facilitate direct competition between T cells with mixed perturbations, aiming to identifying manipulations which robustly augment T cell function. While *in vivo* pooled screens are a crucial step, methods relying on abundance-based, transcriptomic, or standard functional readouts may be limited in predicting long-term cellular fate^31–35^. To link targeted functional manipulations to effects on TF activities and chromatin state, we adapted Spear-ATAC to track diverse modifications in pooled primary murine or human anti-tumor T cells *in vitro* and *in vivo*, predicting a chromatin-level readout may reveal biology to enhance long-term anti-tumor efficacy.

We characterized the chromatin landscape programmed by fixed IL-2 concentrations or a stepwise concentration swap to mimic adoptive transfer, observing IL-2 concentration dictated inferred IL-2 production versus signaling, with ranging stability of TF activities (notably bZIP, RUNX, TCF/LEF). Overall, these data highlight heterogeneity in IL-2 driven programs and suggest strategies to tune T cell transcriptional state.

Moreover, we profiled architecturally distinct CD22-CARs in which modifications to antigen binding affinity and tonic signaling have been shown to drive drastic changes in anti-tumor efficacy in multiple previous reports^2, 9, 36^. Our data highlight the adoptive transfer process as a key determinant of chromatin accessibility, even without antigen engagement. Mapping accessibility changes in our murine IL-2 data to conserved human genomic elements revealed scarcity of IL-2 as a major factor. We also note qualitative and quantitative differences in tonic signal-driven transcriptional signatures in CD4 and CD8 CAR T cells, with greater magnitude Pre-Transfer and Post-Transfer, respectively. This suggests that engineering CD4 and CD8 populations with distinct CAR constructs or perturbations could maximize combined anti-tumor efficacy. Our data further identify early CD8 T cell expansion and CD4/CD8 effector differentiation in T cells expressing CARs which more effectively control CD22^Lo^ leukemia, with the HALL CD22-CAR closely emulating a CD19-CAR response to high antigen levels. Prior studies described positive effects of tonic 4–1BB signaling toward rescuing exhaustion^37^ or inducing the TF *BACH2*, which could be overexpressed in CD28-costimulated CAR T cells to restrain terminal differentiation^36^. Conversely, we report that tonic 4–1BB signaling in SL CD22-CARs alters effector differentiation in both CD4 and CD8 CAR T cells and observe that LL CARs with lower tonic signal confer more physiologic effector programs. Strong effector responses are likely integral to robust targeting of CD22^Lo^ leukemia, a key clinical relapse modality and a requirement for CD22-CARs.

We recently demonstrated overexpression of T-bet in human CD19-CAR T cells with 4–1BB signaling boosted responses to antigen-low leukemia^29^. Refining T-bet mediated enhancements or modulating other pathways like NFκB to synergize with specific CD22-CAR transcriptional programs could prove fruitful. Future studies should also examine stability of cytokine-mediated transcriptional programming of CAR T cells *in vivo*, contributions of tonic signaling from other CARs to T cell differentiation states, and CD4/CD8-specific perturbations.

Our data provide potential insights into mechanisms of Immune Effector Cell-Associated Hemophagocytic Lymphohistiocytosis-Like Syndrome (IEC-HS), a severe clinical toxicity associated with the SASL CD22-CAR. As classical Hemophagocytic Lymphohistiocytosis (HLH) is associated with mutations in effector genes like *PRF1*^38, 39^, we propose altered effector differentiation driven by SL CD22-CARs may contribute to IEC-HS and could be remedied by the non-tonic signaling HALL CD22-CAR. Tracking T cell fates will also be crucial to optimizing therapeutic efficacy and reducing toxicities for promising allogeneic donor-derived and *in vivo* transduction-based therapies with less well-established safety profiles.

In summary, we establish use of scATAC-seq to track *in vitro* and *in vivo* transcriptional programs driven by diverse functional manipulations in barcoded CAR T cell pools. We delineate stable versus transient programs determined by IL-2 concentration during expansion, potentially enabling combinatorial manipulations that necessitate certain cellular states. Finally, while tonic 4–1BB signaling driven by a short linker was previously considered necessary for CD22-CAR function, we show that it restrains effector differentiation and can be circumvented by use of a highly efficacious affinity-matured long linker CAR (HALL). We expect these data and methodologies will inform strategies to tune long-term functional fitness of immune cells, improving outcomes in cancer and other diseases.

## MATERIALS AND METHODS

### Culture of cell lines

EL4 and Jurkat E6–1 cells were obtained from the American Type Culture Collection (ATCC). NALM6-CD22^Lo^ were generated in the Fry laboratory and were originally obtained from the Leibniz Institute DSMZ German Collection of Microorganisms and Cell Cultures GmbH (https://www.dsmz.de/collection/catalogue/details/culture/ACC-128). All lymphocyte cell lines were cultured in Complete RPMI (cRPMI), consisting of RPMI-1640 (Gibco, 11875–093) with 10% (vol/vol) Fetal Bovine Serum (FBS) (Innovative Bioscience, Cat No. 11-01-500), 55μM ß-mercaptoethanol (Sigma-Aldrich, Cat No. M3148–25ML), 2mM L-Glutamine (Gibco, 25030–081) and 500μg/ml (1% vol/vol) of Penicillin/Streptomycin (Gibco, 15140–122). For viral production, the Lenti-X cell line was obtained from Takara Bio (Cat No. 632180) and the Platinum-E cell line was obtained from Cell BioLabs (Cat. No. RV-101). Both viral production cell lines were cultured in Complete DMEM (cDMEM), consisting of DMEM (Gibco, 11965–092) with 10% (vol/vol) FBS, 55μM ß-mercaptoethanol, 2mM L-Glutamine and 500μg/ml (1% vol/vol) of Penicillin/Streptomycin. Viral production cell lines were passaged at least once prior to transfection with viral plasmids. The EL4 cell line stained positive for mouse PD1 and mouse Thy1.2 and negative for human Thy1 and mouse Thy1.1. The Jurkat E6–1 cell line stained positive for human Thy1 and negative for mouse Thy1.2 and Thy1.1. Lenti-X and Platinum-E cell lines were validated morphologically. All cell lines tested negative for *Mycoplasma* contamination using a Universal Mycoplasma Detection Kit (ATCC, Cat No. 30–1012K).

### Preparation of human donor T cells

Healthy human donor blood was obtained from National Jewish Health Blood Donation Core. Blood was processed using Ficoll-Paque Premium (GE Healthcare, Cat No. 17-5442-02) followed by red blood cell lysis in ACK Lysing Buffer (Gibco, Cat No A10492–01) to isolate PBMCs. CD3+ cells were isolated using the MojoSort Human CD3 Selection Kit (BioLegend, Cat No. 480134) and cryopreserved in Freeze Media consisting of 90% FBS and 10% DMSO (Fisher Bioreagents, Cat No. BP231–100) for later use.

### Generation of CAR constructs

Basic murine CAR construction was described previously^22^, with a hCD8A signal peptide, Myc-Tag, anti-human CD19 scFv with (G_4_S)_x3_ linker, mCD28 hinge/transmembrane/costimulatory domain, and mCD3ζ in the MSCV-puro (Clontech) murine retroviral vector, with a porcine teschovirus-1 2A peptide (P2A) followed by mThy1.1 reporter. This original construct was modified to insert a barcode cassette consisting of a 16bp DNA barcode flanked by Nextera sequencing adapters downstream of the mThy1.1 reporter. Basic human CAR construction was described previously, with all CARs consisting of a CD8A signal peptide, respective scFv, CD8A hinge/transmembrane domain, and 4–1BB and CD3ζ signaling domains in the pLenti lentiviral vector^9^. scFvs consisted of: anti-hCD33 (clone huM195, (G_4_S)_x3_ linker), anti-hCD22 (clone m971 (SA) or m971-L7 (HA) with either (G_4_S)_x1_ (SL) or (G_4_S)_x3_ (LL) linker), or anti-hCD19 (clone FMC63, (G_4_S)_x3_ linker). All human CARs contained the P2A, mThy1.1 reporter and a similar barcode cassette as described for the murine CAR. Both DNA constructs contained a WPRE3 sequence^40^ to enhance transgene expression and provide a filler DNA sequence for the nested PCR barcode amplification.

### Barcode generation

The original barcode cassette was generated by assembly of an oligonucleotide pool containing random 16nt sequence (specified as ‘NNNNNNNNNNNNNNNN’) into the plasmid containing murine CAR. Individual colonies were picked and barcode sequence was identified from whole plasmid sequencing (Quintara Biosciences). We selected 6 barcodes with low similarity to other barcodes and low numbers of nucleotide repeats. For the human CAR constructs, a list of indel-correcting 16bp DNA barcodes from the *FreeBarcodes* resource^41^ was filtered using the *create.dnabarcodes()* function^42^ for those with a minimum hamming distance of 13.

### Production of gammaretrovirus and lentivirus

For gammaretrovirus production, 3.5×10^5^ Platinum-E cells were plated in a 6 well plate in cDMEM (see above for recipe) the evening before transfections. The following day, media was replaced with 2.5mL fresh cDMEM, followed by transfection with 1.8μg transfer plasmid and either 0.6μg pCL-Eco (for ecotropic virus used on primary mouse T cells) or 0.6μg pCL-10A1 (for dual pseudotyped amphotropic/ecotropic virus used in barnyard experiment) using TransIT-LT1 Transfection Reagent in 250μL OptiMEM (Gibco, 31985–062). For lentivirus production, 1×10^7^ Lenti-X cells were plated in a T-75 flask in cDMEM the evening before transfections. The following day, media was replaced with 16mL fresh OptiMEM+, consisting of OptiMEM (Gibco, 31985–062) with 5% (vol/vol) FBS, 2mM L-Glutamine and 1mM Sodium Pyruvate (Gibco, 11360–070), followed by transfection with 11.25μg transfer plasmid and pMDLg/pRRE (7.5μg), pMD2.G (3.75μg) and pRSV-Rev (7.5μg) using TransIT-LT1 Transfection Reagent (Mirus Bio, Cat No. 2300) in 2mL OptiMEM (Gibco, 31985–062). Virus-containing supernatant was collected 48hrs later and spun for 5min at 2000xg to remove dead cell particulate before use. All packaging plasmids were obtained from Addgene, with the exception of pCL-10A1 (Novus Biologicals, Cat #NBP2–29542).

### Barnyard experiment transduction

EL4 murine T cell lymphoma and Jurkat E6–1 human T cell leukemia cell lines were cultured in cRPMI as described above. Cell lines were thawed and split once before one round of transduction at 2000xg for 90 minutes with fresh gammaretrovirus and 10μg/mL polybrene, followed by removal of gammaretrovirus and polybrene after the spin and replacement with fresh cRPMI. Cells were transduced with dual pseudotyped amphotropic/ecotropic gammaretrovirus (adding pCL-10A1 envelope instead of pCL-Eco). Transduction efficiency was measured by FACS and then cells were FACS sorted (Lymphocytes>Singlets>Live>mThy1.1+) and captured by scATAC-seq.

### Generation of murine CAR T cells

Mouse CAR T cells were cultured in cRPMI as described above. CD8+ T cells were isolated from 10–16 week old C57BL/6 mouse splenocytes using the MojoSort Mouse CD8 T Cell Isolation Kit (BioLegend, Cat No. 480035). On day 0, T cells were activated with 1μg/mL anti-CD3 and 1μg/mL anti-CD28 overnight. Cells were transduced on day 1 by centrifugation at 2000xg for 90 minutes at 37°C with fresh gammaretrovirus and 10ug/mL polybrene, followed by removal of retrovirus and polybrene after the spin and replacement with fresh media containing the specified amount of recombinant human IL-2, either 10IU/mL (‘Hi’) or 100IU/mL (‘Lo’) (National Cancer Institute). Transduction was repeated on the following day (day 2), and then cells were removed from anti-CD3/anti-CD28 stimulation and plated at 1×10^6^ cells/mL in fresh media containing IL-2. On day 3 and 4, cells were replated at 1×10^6^ cells/mL in fresh media containing IL-2. For the ‘Swap’ condition, IL-2 concentration was changed from 100IU/mL to 10IU/mL. Cells were FACS sorted (Lymphocytes > Singlets > Live > CD8+ > mThy1.1+) and captured for scATAC-seq on day 5, 16 hours after the IL-2 swap (or media replacement for the Hi and Lo conditions).

### Generation of human CAR T cells

CD3+ human T cells were cultured in Human T Cell Expansion Media (hTCEM), consisting of AIM-V (Gibco, Cat No 12055–091) supplemented with 5% FBS and 10mM HEPES (Gibco, 15630–080). On day 0, T cells were thawed and activated with Dynabeads Human T cell Expander CD3/CD28 (Gibco, Cat No. 11141D) at a 3:1 bead:cell ratio at 1×10^6^ cells/mL, in hTCEM supplemented with 40IU/mL IL-2 and cultured for 48 hours. On day 2, T cells were counted and transduced at 2×10^6^ cells per well in a 6 well plate, with 10μg/mL Protamine Sulfate (Millipore-Sigma, Cat No. P4020–1G), spinning at 1000xg for 2.5 hours at 32°C. On day 3, T cells were transduced again with fresh virus. On day 4, beads were removed, and cells were plated in fresh hTCEM supplemented with 100IU/mL recombinant human IL-2 at 0.5×10^6^ cells/mL. On day 6, cells were replated at 0.5×10^6^ cells/mL with fresh hTCEM and 100IU/mL IL-2, and a small aliquot was taken to check transduction efficiency. On day 8, cells were counted with a small aliquot taken for FACS sorting (Lymphocytes > Singlets > Live > CD4+ or CD8+ > mThy1.1+) and scATAC-seq capture (Pre-Transfer timepoint), with the rest cryopreserved in Freeze Media as described above.

### Nalm6 xenograft *in vivo* studies

NOD-Scid-Gamma (‘NSG’, NOD.Cg-*Prkdc*^*scid*^*Il2rgtm1Wjl*/SzJ; Strain #: 005557, Jackson Laboratories) were obtained commercially and then bred in-house. For xenograft studies, between 4 and 9 male or female NSG mice (up to 20 weeks of age, same gender in a single experiment) received 1×10^6^ NALM6-CD22^Lo^ leukemia on day −3, followed by 1×10^6^ total CAR+ cells (1.67×10^5^ of each CAR, pooled equally) on day 0. Leukemia burden was monitored before CAR injection (day −1), on day 4, and on day 7 before marrow harvest, using the Xenogen In Vivo Imaging System (IVIS) Spectrum (Caliper Life Sciences) after intraperitoneal injection with D-Luciferin Potassium Salt (Revvity, Cat No. 122799A-5). Total flux (photons/s) was measured for each mouse using Living Image software (Revvity, v4.8.2). On day 7, bone marrow was harvested, filtered through a 70μm strainer, and red blood cells were lysed using ACK Lysing Buffer. Cells were then filtered again, and human cells were enriched using MojoSort Mouse CD45 Nanobead (BioLegend, Cat No. 480028) depletion of mouse bone marrow. Cells were stained and FACS sorted (Lymphocytes > Singlets > Live > CD45+/GFP− > CD4+ or CD8+ > mThy1.1+) for scATAC-seq capture (Post-Transfer timepoint). Xenograft animal studies were conducted at University of Colorado Anschutz Medical Campus in accordance with an animal protocol approved by the Institutional Animal Care and Use Committee (IACUC, Protocol 751).

### Flow cytometry and FACS sorting

Flow cytometric analysis was performed on an A5 Flow Cytometer (BD Biosciences). For the barnyard experiment in cell lines and the IL-2 experiment in primary mouse T cells, transduction was detected by Myc-Tag+(CAR+)/mThy1.1+ double positive cells. For the human CAR T cell experiments, transduction was detected by Protein-L+/mThy1.1+ double positive cells (to compare geometric mean fluorescence intensity (GMFI) across all CAR constructs) and by Protein-Fc+/mThy1.1+ double positive cells (using either CD19, CD22, or CD33 Protein-Fc as a direct readout of antigen binding for each CAR). Both readouts showed similar detection; Protein-Fc/mThy1.1+ cells was used as the final readout of CAR+ cells for downstream experiments. Cells were stained and sorted in FACS Buffer consisting of PBS (Fisher Bioreagents, Cat No. BP2438–4) with 2mM EDTA (Fisher Bioreagents, Cat No. BP2482–100) and 2% FBS. Sorted cells were collected into 1.5mL tubes containing Sort Buffer consisting of PBS with 0.04% UltraPure BSA (Invitrogen, Cat. No. AM2618). Dead cells were excluded using eBioscience Fixable Viability Dye (Invitrogen, Cat. No. 65-0865-18). NALM6 leukemia was identified by GFP. Flow cytometry data was analyzed and plotted using FlowJo (Becton Dickinson & Company, v10.10.0). Some graphs quantifying flow cytometry data were generated using Prism (GraphPad, v10.4.1).

### scATAC-seq capture and library preparation

All steps prior to 10X scATAC-seq capture were performed on ice or in a microcentrifuge cooled to 4°C. For in vivo assays, cells were pooled before transfer and sorted into one tube upon harvest. For *in vitro* assays, cells from each condition were sorted, analyzed for live cell counts via 0.4% Trypan Blue staining on the Countess 3 (ThermoFisher), and pooled at equal numbers. After pooling, cells were washed once in cold PBS and then lysed in Lysis Buffer (Nuclease-Free H_2_O with 10 mM Tris-HCl, pH 7.5, 10 mM NaCl, 3 mM MgCl2, 0.1% NP40, 0.1% Tween20, and 0.01% Digitonin) for the following times: 5 mins (cell lines), 3 mins (*in vitro* primary cells), 3 mins 45 seconds (Post-Transfer primary cells). Cells were then resuspended in 1X Nuclei Buffer and analyzed for nuclei count and lysis efficiency via 0.4% Trypan Blue staining on the Countess 3 (ThermoFisher), with a typical lysis efficiency of >95%. Cells were then diluted to ≤2.4×10^6^ cells/mL for capture of ~8000 nuclei in a single 10X capture well, following manufacturer protocols for Chromium Next GEM Single Cell ATAC Reagent Kits v2 (10X Genomics, User Guide CG000496). Two modifications were required to this protocol for barcode enrichment, as described previously^21^: first, 1.32μL of 50μM spike-in barcode amplification oligonucleotide (Oligo 1032, see Supplementary Table 1) was added to the GEM reaction master mix (step 2.1a in 10X protocol); second, the PCR following GEM generation was increased from 12 to 15 cycles (step 2.5a in 10X protocol). Libraries were prepared per manufacturer specifications and analyzed using the TapeStation (Agilent) and Qubit Fluorometer (ThermoFisher).

### Barcode enrichment and library preparation

For specific sequences of all primers used, see Supplementary Table 1. Barcodes were enriched from the final scATAC library with the following ‘Barcode Enrichment’ PCR:

**Table:** 

PCR Mix		Thermocvcling
100uL	NEB Q5 2x MasterMix	98°C 1min
2uL	Oligo 908	98°C 10sec }
2uL	Oligo 1033	67°C 15sec } X cycles (see *Note below)
1.5uL	Diluted scATAC Library (5ng total)	72°C 1min }
94.5uL	H_2_O	72°C 1min
		10°C ∞

* Note: The following cycle numbers were optimized and used for the barcode enrichment PCR: 16 cycles (retroviral transductions), 25 cycles (lentiviral transductions). We predict that our lentiviral protocol, optimized for lower vector insertion copy numbers per cell, required a greater number of cycles for amplification of the barcode fragments from tagmented chromatin.

The Barcode Enrichment PCR product was cleaned up using the DNA Clean and Concentrator-5 Kit (Zymo Research, D4004). Purified DNA was then run on 2% E-Gel SizeSelect II Agarose Gels (Invitrogen, Cat No. G661012) to select the enriched barcode DNA. Unique i7 indices were added to each individual barcode library using custom indexing primers by running the following ‘Barcode Indexing’ PCR:

**Table:** 

PCR Mix		Thermocvcling
100uL	NEB Q5 2x MasterMix	98°C 1min
2uL	Oligo 908	98°C 10sec }
2uL	Oligo 92X*	67°C 15sec } 13 cycles
1.5uL	Diluted E-Gel Enrichment PCR (12 pg)	72°C 1min }
94.5uL	H_2_O	72°C 1min
		10°C ∞

* Oligo 92X indicates any oligonucleotide from series 925–928, which are identical except for the 8bp P7 index which allows pooling of indexed samples.

The Barcode Indexing PCR product was taken directly into a 1.1x left side size selection with SPRIselect Beads (Beckman Coulter, Cat No. B23318) using manufacturer protocols for isolation of final barcode library. DNA quality and size distribution was monitored by Tapestation after each step, and by Tapestation and Qubit analysis after the final SPRI selection.

### Sequencing

Final scATAC and barcode libraries were pooled to 10nM concentration for sequencing. Final concentrations for individual libraries in the pool were calculated to achieve a target sequencing depth of 40,000 reads/nuclei (scATAC library) and 1,000 reads/nuclei (barcode library), and pooled libraries were sequenced on an Illumina NextSeq 2000 at the National Jewish Health Genomics Facility (Research Resource Identifier – RRID: SCR_023051).

### Alignment

Raw sequencing data was processed into fastq files using *cellranger-atac* (10X Genomics, v2.1.0), using the *mkfastq* function, and reads were aligned to the Genome Reference Consortium human hg38 and/or murine mm10 reference genomes using the *count* function.

### Barnyard analysis

In addition to the barcode-transduction experiment described above, scATAC was performed on a mixed pool of untransduced EL4 and Jurkat cells to verify barcode distribution did not change fragment size distribution (Figure S1A). Barnyard analysis is n=1 experiment each for the untransduced and barcode-transduced pools. After alignment, barcodes were mapped in R using custom functions and functions adapted from the Spear-ATAC manuscript^21^. Reads were mapped to a combination mm10/hg38 reference genome. Quality control values, number of reads mapping to mouse and human genomes, and species assignments were taken directly from cellranger outputs.

### Spear-ATAC Analysis: General

The ArchR R package (https://www.archrproject.com, v1.0.3)^43^ was used for all other downstream processing and analysis of scATAC-seq data in primary murine and human T cells. General analysis parameters were followed for both murine and human datasets, with several modifications for the human data as noted, which were either generally more appropriate or accounted for the increased experimental complexity associated with additional replicates and timepoints. We preprocessed the data, reading in barcodes using *getValidBarcodes()* followed by creation of Arrow files using *createArrowFiles()* to read accessible genomic fragments, filter to include TSS enrichment >10 and >10,000 fragments for murine cells (TSS enrichment >8 and >10,000 fragments for human cells), creating a genome-wide TileMatrix using 500bp bins, and creating a GeneScoreMatrix using the appropriate reference genome. An ArchR project was then created using and *ArchRProject()*. We then filtered to include cells with assigned barcodes, reading in all fragments with valid cell barcodes and viral barcodes before assigning each cell a viral barcode using cutoffs of 0.95 for ‘barcode specificity’ (proportion of barcode reads matching barcode with most reads) and 100 reads for log_10_(# of barcode reads) for barcode with most reads, with the final barcode assignment being the barcode with the most reads. Dimensionality reduction was performed using ArchR’s native Iterative Latent Semantic Indexing implementation^44–46^ via *addIterativeLSI()*, with *LSImethod* = 3 (logtf-logidf), *dimsToUse* = 1:13, *useMatrix* = TileMatrix, with *varFeatures* = 25,000 for the murine data (increased to 50,000 for human data). Harmony batch correction was run on human data to correct for pooling biological donors^47^, using the *addHarmony()* function with *groupBy* = Replicate. Clusters were called using Seurat^48^, and UMAP added using *addUMAP()* with the IterativeLSI-reduced dimensions (Harmonized IterativeLSI for human data). Finally, peaks were called on Seurat clusters, using *addGroupCoverages()* (minReplicates increased to 3 for human), and *addReproduciblePeakSet()* with *method* = q, *cutoff* = 0.005 (0.001 for human), *peaksPerCell* = 500, *maxPeaks* = 75,000 (65,000 for human), extendSummits = 250, with MACS2^49^ before creating a PeakMatrix using *addPeakMatrix()*. Motif annotations were added using *addMotifAnnotations()* with the vierstra archetype motif set^50^, and motif deviations archetypeMatrix was added using *addDeviationsMatrix()*.

### Spear-ATAC Analysis: Murine CAR T cell specific analyses

Multidimensional Scaling (MDS) dimensionality reduction was used to analyze global similarity in chromatin accessibility profile between pseudobulked cells from each barcode replicate and by condition. Murine IL-2 culture condition analysis is n = 1 experiment with n = 2 independent barcoded cultures per culture condition.

### Spear-ATAC Analysis: Human CAR T cell specific analyses

Coreceptor assignment cutoffs were designated by visualizing *CD4*, *CDA*, *CD8B* gene scores, and timepoint/coreceptor subsets were created using *subsetArchRProject()*. To create the motif enrichment heatmap in Figure 3, we defined differentially accessible peaks for each cluster using *getMarkerFeatures()* with *testMethod* = “wilcoxon” and *getMarkers()* with false discovery rate ≤ 0.01 and the absolute value of log_2_(fold-change) ≥ 1 before plotting with *plotMarkerHeatmap()*. Motifs enriched within differential accessible peaks were then defined using *peakAnnoEnrichment()* with false discovery rate ≤ 0.01 and the absolute value of log_2_(fold-change) ≥ 0.5, before plotting using plotEnrichHeatmap() with n = 6 maximum motifs per cluster and *cutoff* = 20 for minimum adjusted p-value to be included in the heatmap. Comparisons of accessibility for genetic loci tracks were made using either the previously defined differentially accessible peaks for each cluster or by defining differentially accessible peaks between all Pre-Transfer and Post-Transfer cells, both with false discovery rate ≤ 0.1 and log_2_(fold-change) ≥ 0.5. Coordinates for the TF activity summary plots to visualize motifs associated with either CD22-CAR affinity or linker were calculated as the difference between ChromVAR^27^ motif-associated chromatin accessibility z-scores for the comparisons as indicated on each axis. Points closer to the y = x diagonal line are more directly biased by affinity or linker as indicated, while points away from the diagonal exhibit preferential bias by either the x-axis or y-axis comparison. Cutoff value of >0.5 in any direction was applied for Post-Transfer data (Figure 4) and >0.3 for pre-transfer data (Figure S4). Human CAR T cell analysis is n = 2 experiments with independent biological human donors.

### Interspecies genomic mapping

The University of California Santa Cruz Genome Browser ‘Liftover’ tool^51^ was used to compare the relative chromatin accessibility of human T cell populations at homologous regions between mm10 and hg38 reference genomes. The regions used were the sets of peaks that were differentially accessible between mouse T cells cultured in ‘Hi’ and ‘Lo’ IL-2, mapping between mm10 and hg38 reference genomes. Default settings were used, with the exception of the “minimum ratio of bases that must remap” setting, which was changed to 0.5 to increase stringency. Liftover for peaks down in Hi compared to Lo resulted in 3998 converted peaks and 2894 failing to meet cutoffs, while up in Hi compared to Lo resulted in 4871 converted peaks and 3606 failing to meet cutoffs. Liftover comparisons were exported as bed files and peak annotations were added to the ArchR project using *addDeviationsMatrix()* before UMAP visualization.

## Supplementary Material

1

## Data Availability

All data is available from the corresponding author upon reasonable request. Genomics data is uploaded to the Gene Expression Omnibus (GEO) under accession number GSE331391.
