# Supplementary material for: Single-cell genomics links targeted functional manipulations to efficacy-associated chromatin signatures in CAR T cells
Source: Res Sq. 2026 Jun 29:rs.3.rs-9859689. Preprint. [Version 1] doi: 10.21203/rs.3.rs-9859689/v1 (PMC13345528; doi:10.21203/rs.3.rs-9859689/v1)
Supplement: 1 [file NIHPPRS9859689V1-supplement-1.pdf]

EXTENDED DATA

Figure S1

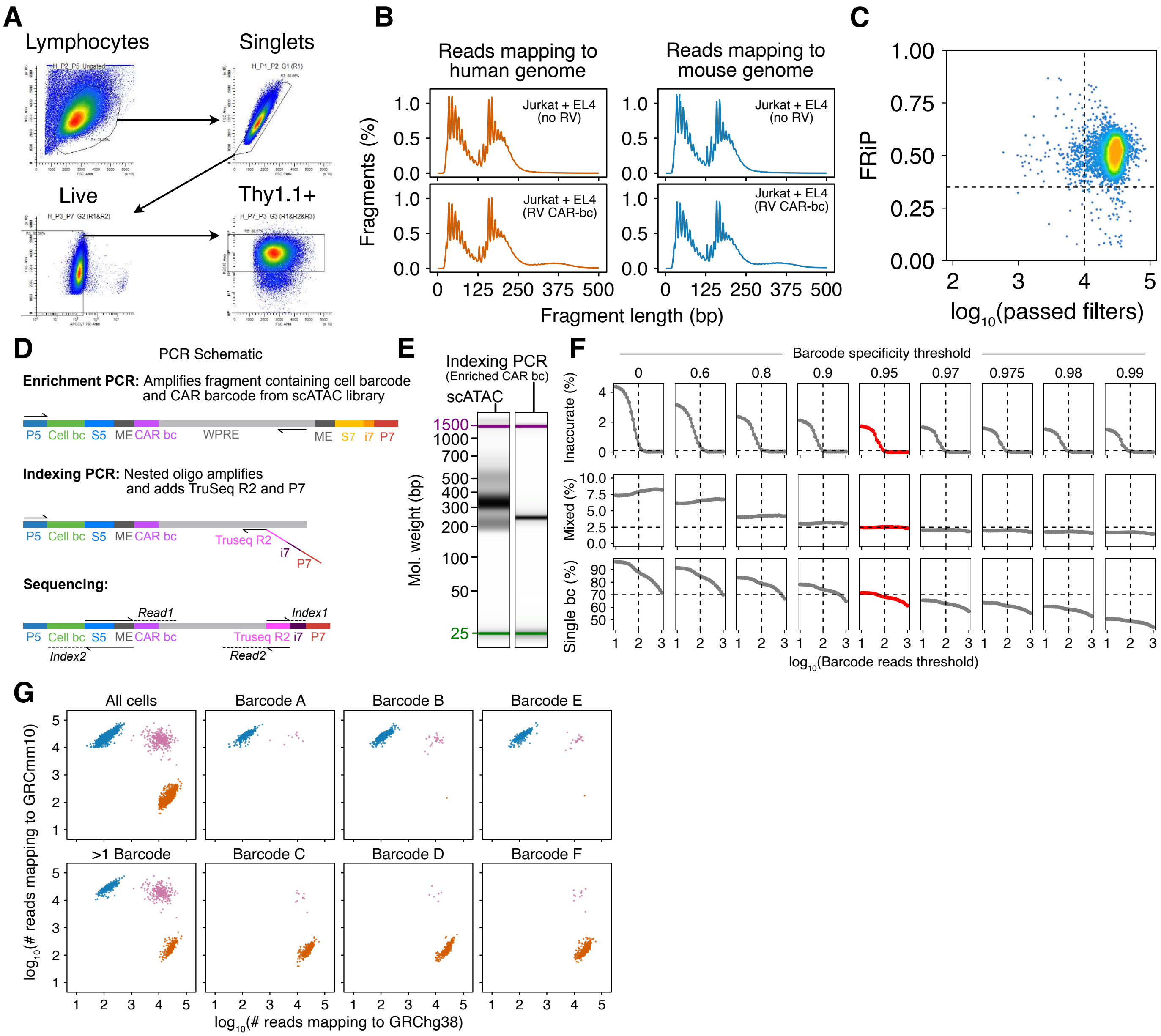

Figure S1: Further characterization of species-associated barcoding to optimize *in silico* filtering parameters

S1A: FACS gating schematic for the sorting of barcoded EL4 and Jurkat cell lines (Lymphocytes>Singlets>Live>mThy1.1+). **S1B:** Distribution of fragment sizes for reads mapping to human GRChg38 (left column) and mouse GRCmm10 (right column) reference genomes for untransduced EL4 and Jurkat pool (top row) and barcoded CAR-transduced EL4 and Jurkat pool (bottom row). All data for **S1C-S1G** is from the barcoded CAR-transduced EL4 and Jurkat pool. **S1C:** Fraction of Reads in Peaks (FRiP) by $\log_{10}$(# of reads passing filters). **S1D:** PCR Schematic: Two step nested PCR approach to 1) enrich for DNA fragments containing the introduced DNA barcode from the final scATAC library and 2) add a sample index and TruSeq adapter sequence to create the final 'barcode library' to be sequenced alongside the scATAC library. **S1E:** Agilent Tapestation analysis showing fragment size distribution for scATAC library (left) and final 'barcode library' after sample indexing PCR (right). **S1F:** Plots showing % of included cells with inaccurate species assignments (top row), containing mixed barcodes (middle row), and containing a single barcode (bottom row) on the y-axis, plotted by the barcode reads threshold ($\log_{10}$(# of barcode reads matching barcode with most reads)) for a range of 'barcode specificity' thresholds (proportion of barcode reads matching barcode with most reads) across each row. **S1G:** Plots of $\log_{10}$(# reads mapping to GRCmm10) by $\log_{10}$(# reads mapping to GRChg38). "All cells" plot contains all cells prior to application of 100 reads sensitivity and 0.95 specificity filtering thresholds, while ">1 Barcode" plot contains cells that had more than one barcode prior to filtering. Plots for each barcode contain cells which were assigned the indicated barcode after filtering cutoffs were applied.

Figure S2

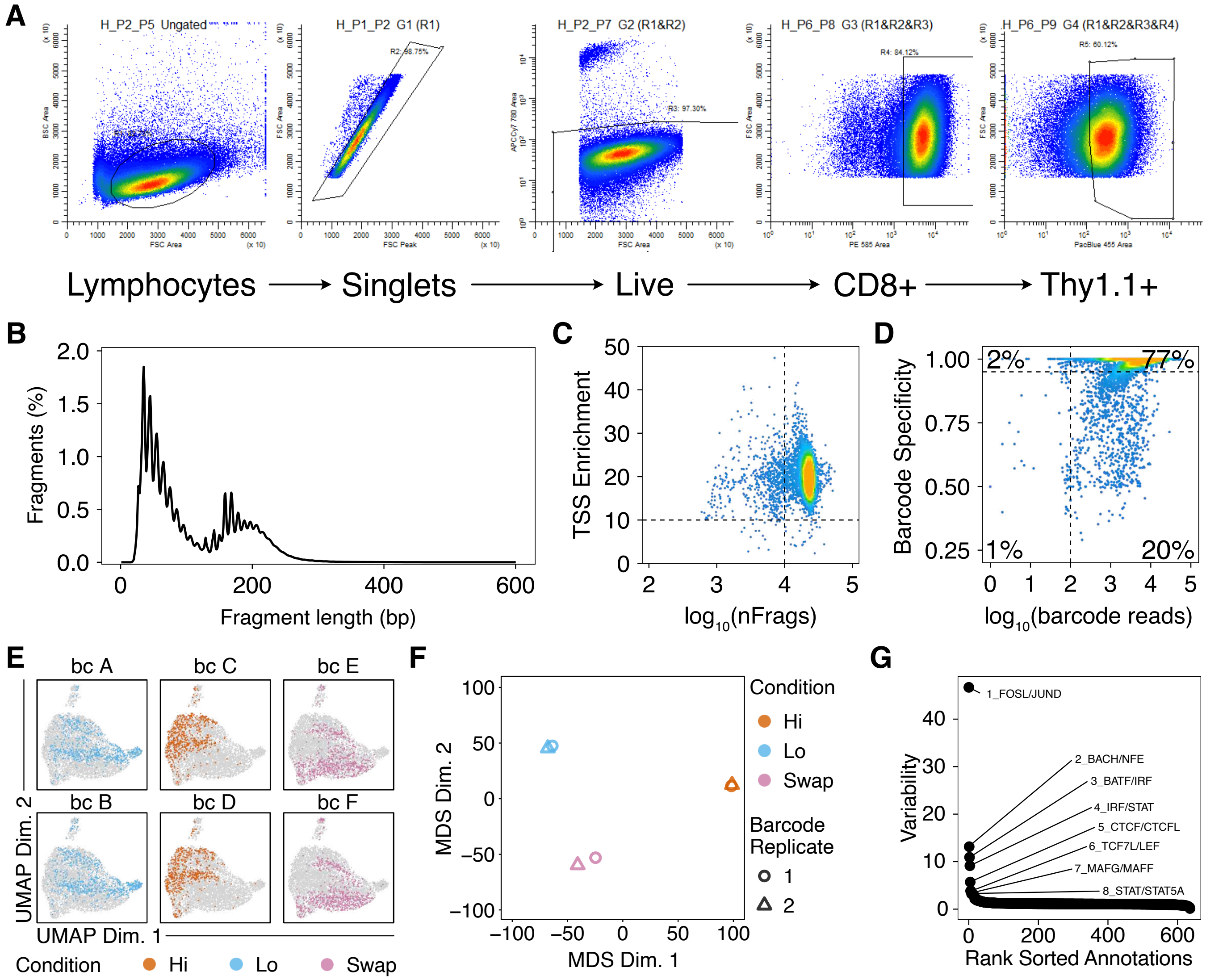
A
Lymphocytes → Singlets → Live → CD8+ → Thy1.1+
B
Fragments (%)
Fragment length (bp)
C
TSS Enrichment
log10(nFrags)
D
Barcode Specificity
2%
77%
1%
20%
log10(barcode reads)
E
bc A
bc C
bc E
bc B
bc D
bc F
UMAP Dim. 2
UMAP Dim. 1
Condition
Hi
Lo
Swap
F
MDS Dim. 2
MDS Dim. 1
Condition
Hi
Lo
Swap
Barcode Replicate
1
2
G
Variability
Rank Sorted Annotations
1_FOSL/JUND
2_BACH/NFE
3_BATF/IRF
4_IRF/STAT
5_CTCF/CTCFL
6_TCF7L/LEF
7_MAFG/MAFF
8_STAT/STAT5A

Figure S2: Quality control and characterization of IL-2 culture condition-associated barcoding of primary murine CAR T cells

S2A: FACS gating schematic for the sorting of barcoded murine CAR T cells (Lymphocytes > Singlets > Live > CD8+ > mThy1.1+). **S2B:** Distribution of fragment sizes. **S2C:** $\log_{10}$(number of fragments) by TSS enrichment. **S2D:** $\log_{10}$(# of barcode reads matching barcode with most reads) by barcode specificity (proportion of barcode reads matching barcode with most reads), with filtering cutoffs indicated. **S2E:** UMAP visualization of cells assigned each barcode, colored by IL-2 condition. **S2F:** Multidimensional Scaling (MDS) dimensionality reduction indicating global similarity in chromatin accessibility profile between pseudobulked cells from each barcode replicate and by condition. Coloring indicates IL-2 condition. Shape indicates barcode replicate, where replicate 1 is barcodes A, C and E for Lo, Hi and Swap conditions, and replicate 2 is B, D and F for Lo, Hi and Swap conditions. **S2G:** Ranked list of most variable ChromVAR motif-associated chromatin accessibility z-scores between all conditions.

Figure S3

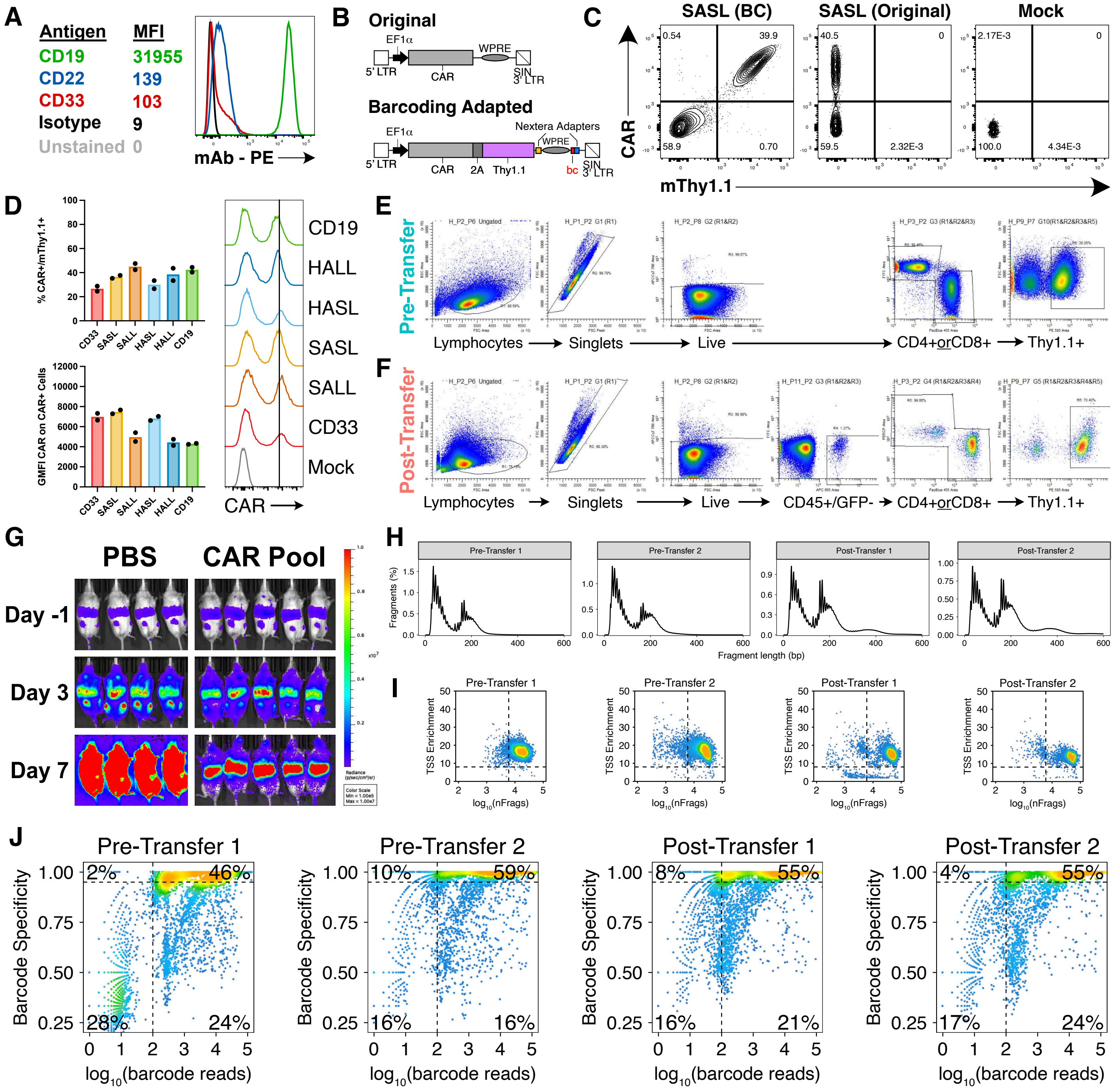
A
Antigen MFI
CD19 31955
CD22 139
CD33 103
Isotype 9
Unstained 0
mAb - PE
B
Original
EF1α
5' LTR
CAR
WPRE
SIN 3' LTR
Barcoding Adapted
Nextera Adapters
2A
Thy1.1
bc
C
SASL (BC)
SASL (Original)
Mock
CAR
mThy1.1
D
% CAR+/mThy1.1+
GMFI CAR on CAR+ Cells
CD33 SASL SALL HASL HALL CD19
CD19
HALL
HASL
SASL
SALL
CD33
Mock
CAR
E
Pre-Transfer
Lymphocytes → Singlets → Live → CD4+orCD8+ → Thy1.1+
F
Post-Transfer
Lymphocytes → Singlets → Live → CD45+/GFP- → CD4+orCD8+ → Thy1.1+
G
PBS
CAR Pool
Day -1
Day 3
Day 7
H
Pre-Transfer 1
Pre-Transfer 2
Post-Transfer 1
Post-Transfer 2
Fragments (%)
Fragment length (bp)
I
TSS Enrichment
log10(nFrags)
J
Pre-Transfer 1
Pre-Transfer 2
Post-Transfer 1
Post-Transfer 2
Barcode Specificity
log10(barcode reads)

Figure S3: Quality control and characterization of CD22-CAR architecture-associated barcoding of primary human T cells

S3A: Flow cytometric analysis of CD19, CD22 and CD33 antigen expression on NALM6-CD22^{Lo} leukemia, compared to unstained and isotype-stained controls. All antigens were stained with PE-conjugated antibodies, and used the same PE-conjugated isotype control. Mean fluorescence intensity (MFI) for each antigen is indicated on the right. **S3B:** Schematic: Original CAR constructs (top) in pLenti backbone contain 5'UTR, EF1a promoter, CAR transgene, WPRE3 and self-inactivating (SIN) 3'LTR. Barcoding adapted constructs in pLenti backbone contain 5'UTR, EF1a promoter, CAR transgene, P2A, murine Thy1.1 reporter, barcode cassette containing WPRE3 and 16bp DNA barcode flanked by Nextera sequencing adapters, and self-inactivating (SIN) 3'LTR. **S3C:** Flow cytometry showing SASL CAR expression (CD22 Protein-Fc) by mThy1.1 reporter expression for the Barcoding Adapted CAR ("BC"), original CAR compared to Mock (untransduced) T cells. **S3D:** Transduction efficiency (top, % CAR+/mThy1.1+) and geometric mean fluorescence intensity (GMFI, bottom) on CAR+ T cells from both donors. Representative flow plots shown to the right. To facilitate equivalent CAR molecule GMFI detection independent of binding affinity or detection reagent, CAR T cells were stained with Protein L. **S3E-F:** FACS gating schematic for the sorting of barcoded human CAR T cells at Pre-Transfer (Lymphocytes > Singlets > Live > CD4+ <u>or</u> CD8+ > mThy1.1+) **(E)** and Pre-Transfer (Lymphocytes > Singlets > Live > CD45+/GFP- > CD4+ <u>or</u> CD8+ > mThy1.1+) **(F)** timepoints. **S3G:** Bioluminescence imaging showing leukemia burden over time in NSG mice given 1e6 NALM6-CD22^{Lo} on Day -3 before treatment at Day 0 with PBS (left) or 1e6 pooled CAR T cells (1.67e5 of each CAR, shown in Figure 3D). **S3H-J** show the following QC metrics for both timepoints (Pre-Transfer, Post-Transfer) and both donors (1, 2): **S3H:** Distribution of fragment sizes. **S3I:** $\log_{10}$(number of fragments) by TSS enrichment. **S3J:** $\log_{10}$(# of barcode reads matching barcode with most reads) by barcode specificity (proportion of barcode reads matching barcode with most reads), with filtering cutoffs indicated.

Figure S4

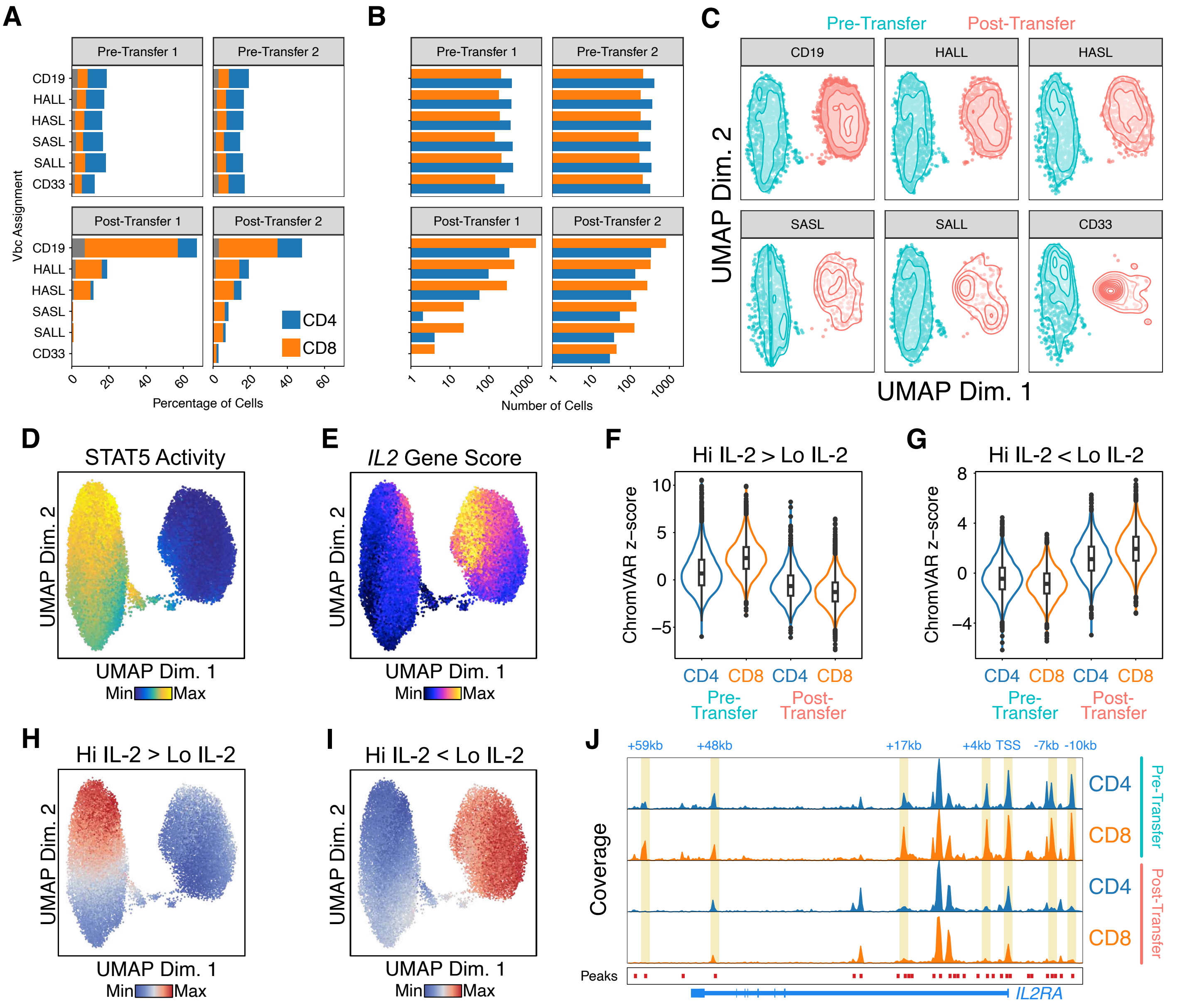
A
Pre-Transfer 1
Pre-Transfer 2
Post-Transfer 1
Post-Transfer 2
CD19
HALL
HASL
SASL
SALL
CD33
Vbc Assignment
CD4
CD8
Percentage of Cells
B
Number of Cells
C
Pre-Transfer
Post-Transfer
UMAP Dim. 2
UMAP Dim. 1
D
STAT5 Activity
Min
Max
E
IL2 Gene Score
F
Hi IL-2 > Lo IL-2
ChromVAR z-score
CD4
CD8
Pre-
Transfer
Post-
Transfer
G
Hi IL-2 < Lo IL-2
H
I
J
+59kb
+48kb
+17kb
+4kb
TSS
-7kb
-10kb
Coverage
Peaks
IL2RA

Figure S4: Temporal changes occurring from Pre- to Post-Transfer and murine-to-human IL-2 liftover analysis in primary human CAR T cells

S4A-B: Quantification of barcode and coreceptor assignments for pooled T cells at Pre-Transfer (top row) and Pre-Transfer (bottom row) timepoints, separated by donor replicates. Plots quantify proportions **(A)** and absolute numbers **(B)**. **S4C:** UMAP visualization of all cells, separated by CAR assignment and colored by timepoint.

S4D-E: UMAP visualization of pooled cells from both timepoints, colored by STAT5 motif-associated chromatin accessibility (z-score) **(D)**, IL2 gene score **(E)**. **S4F-G:** Violin plots of ChromVAR z-scores quantifying enrichment of groups of peaks in human CAR T cell coreceptor/timepoint subgroups which were either more accessible in murine cells cultured in Hi compared to Lo IL-2 **(F)** or more accessible in murine cells cultured in Lo compared to Hi IL-2 **(G)**. **S4H-I:** UMAP visualization of the data in S4F (for panel **H**), or the data in S4G (for panel **I**). **S4J:** Genomic chromatin accessibility tracks surrounding the *IL2RA* locus for cells pseudobulked and stratified by timepoint (Pre-Transfer/Post-Transfer) and coreceptor (CD4/CD8). Boxes indicate peaks which were differentially accessible between timepoints at putative or established regulatory elements surrounding each gene locus. Distance from nearest TSS indicated.

Figure S5

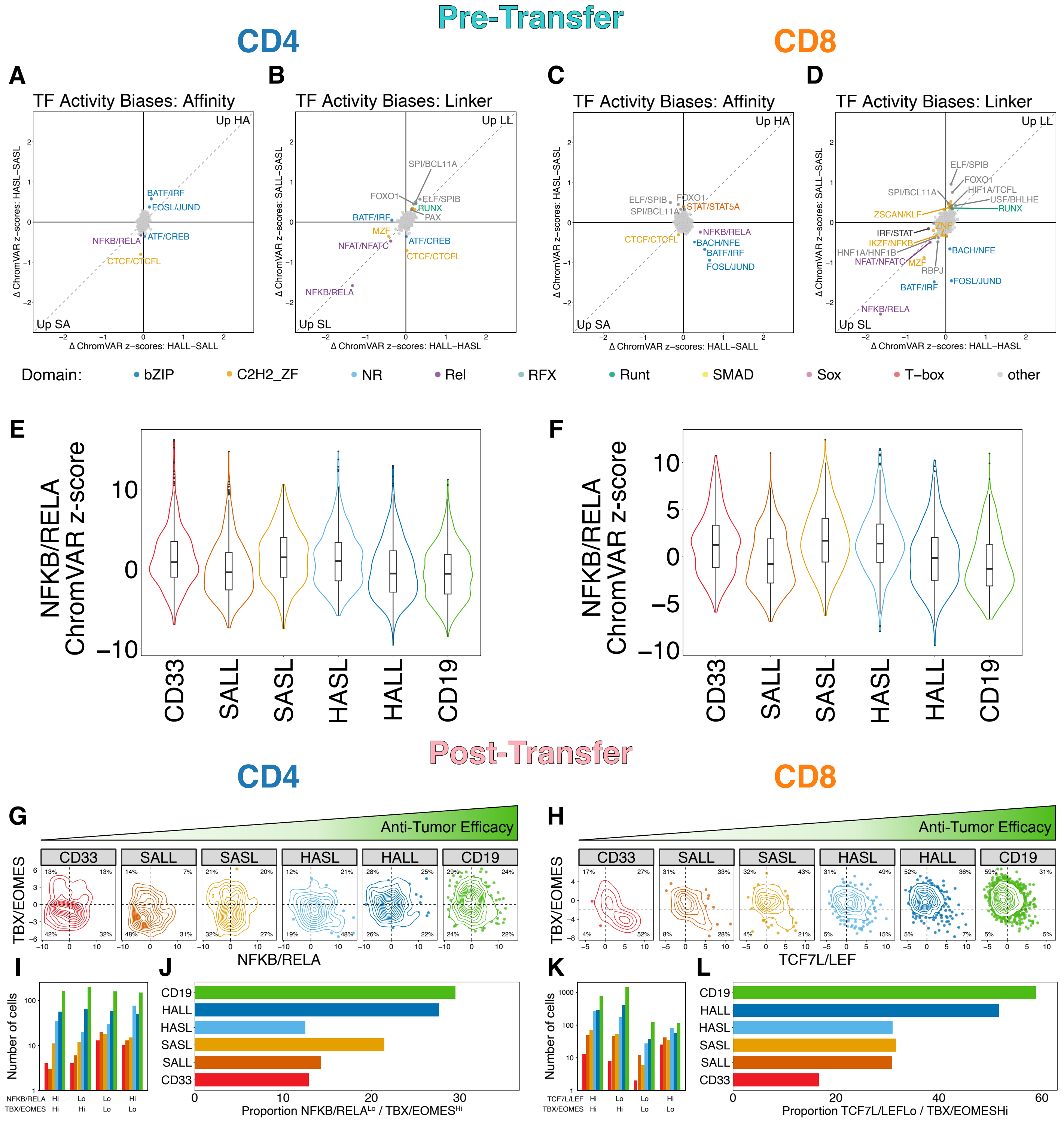

Pre-Transfer
CD4
CD8
A
B
C
D
TF Activity Biases: Affinity
TF Activity Biases: Linker
Up HA
Up LL
Up SA
Up SL
Δ ChromVAR z-scores: HASL–SASL
Δ ChromVAR z-scores: HALL–SALL
Δ ChromVAR z-scores: SALL–SASL
Δ ChromVAR z-scores: HALL–HASL
Domain:
bZIP
C2H2_ZF
NR
Rel
RFX
Runt
SMAD
Sox
T–box
other
E
F
NFKB/RELA ChromVAR z-score
CD33
SALL
SASL
HASL
HALL
CD19
Post-Transfer
G
H
Anti-Tumor Efficacy
TBX/EOMES
NFKB/RELA
TCF7L/LEF
I
J
K
L
Number of cells
Proportion NFKB/RELA^{Lo} / TBX/EOMES^{Hi}
Proportion TCF7L/LEFLo / TBX/EOMESHi

Figure S5: CD22-CAR architectures drive distinct NFκB, TCF/LEF and effector-associated transcriptional programs prior to transfer and during the anti-leukemia response

Data on left side of page (panels A, B, E, G, I, J) are from CD4 CAR T cells and data on right side of page (panels C, D, F, H, K, L) are from CD8 CAR T cells. **S5A-D:** CD22-CAR architectural biases in transcriptional activity for transcription factors binding the indicated motifs, driven by either CD22-CAR affinity (**A**, **C**) or linker (**B**, **D**) at the Pre-Transfer timepoint. Coordinates are calculated as the difference between ChromVAR motif-associated chromatin accessibility z-scores for the comparisons as indicated on each axis. Points closer to the y = x diagonal line are more directly biased by affinity or linker as indicated, while points away from the diagonal exhibit preferential bias by either the x-axis or y-axis comparison. Cutoff value of >0.3 in any direction was applied. **S5E-F:** Comparisons of ChromVAR motif-associated chromatin accessibility z-scores for NFκB, for all cells assigned each barcode at the Pre-Transfer timepoint. **S5G-H:** Pairwise comparisons of ChromVAR motif-associated chromatin accessibility z-scores for all cells assigned each barcode for the indicated set of motifs. **S5I:** Quantifications of total cell numbers in each quadrant of S5G, stratified by CAR. **S5J:** Proportions of each CAR in the indicated quadrant of S5G. **S5K:** Quantifications of total cell numbers in each quadrant of S5H, stratified by CAR. **S5L:** Proportions of each CAR in the indicated quadrant of S5H.

Table S1

Oligo #	Name	Sequence	Length (bp)	Description
908	SI-PCRprimerB	AATGATACGGCGACCACCGAGA	22	Same as 10x ATAC SI-PCR primer b
925	PCR 2 primer 1 Spear ATAC	CAAGCAGAAGACGGCATACGAGAT ACGAGTAG GTGACTGGAGTTCAGACGTGTG	56	Spear ATAC - PCR 2 p7 indexed primer 1
926	PCR 2 primer 2 Spear ATAC	CAAGCAGAAGACGGCATACGAGAT ACCCGCAC GTGACTGGAGTTCAGACGTGTG	56	Spear ATAC - PCR 2 p7 indexed primer 2
927	PCR 2 primer 3 Spear ATAC	CAAGCAGAAGACGGCATACGAGAT AATTGAAC GTGACTGGAGTTCAGACGTGTG	56	Spear ATAC - PCR 2 p7 indexed primer 3
928	PCR 2 primer 4 Spear ATAC	CAAGCAGAAGACGGCATACGAGAT ACGCGGAA GTGACTGGAGTTCAGACGTGTG	56	Spear ATAC - PCR 2 p7 indexed primer 4
1032	Thy1.1 Spike-In Oligo	CCTGGACTTCATTTCTCTGTGATAA	25	Thy1.1-Specific Spike-In Oligo
1033	Enrich V2 JSB364 2	/5BiosG/GTGACTGGAGTTCAGACGTGTGCTCTTCCGATCTTTAGTTCTTGCCACGGCGGAAC	64	Barcode Enrichment Oligo. Specific to WPRE3.

*Note: Oligo # 1033 has 5' biotinylation to facilitate alternative enrichment strategies using Streptavidin pulldown as used in the original Spear-ATAC paper if necessary.
8nt sequence in middle of 92X primers is p7 index.

Table S1: List of oligonucleotides used in this study

Table S2

Barcode ID	Sequence	Note
A	CGCAATGCTGGCGCCT	Used in Fig 1, 2, S1, S2
B	GAACGCAGTGTTAACG	Used in Fig 1, 2, S1, S2
C	TGGAATACCCGACAGT	Used in Fig 1, 2, S1, S2
D	GACTTAGGTGTAGATC	Used in Fig 1, 2, S1, S2
E	GCTTTTCACGTAGTCT	Used in Fig 1, 2, S1, S2
F	CGTGTACTAAAGGCTC	Used in Fig 1, 2, S1, S2
1	GGCAGGTGATCGCAAT	Used in Fig 3, 4, S3, S4, S5
2	ATTATTGGCGTTGACC	Used in Fig 3, 4, S3, S4, S5
3	CTGGAGAAGAGTTGGC	Used in Fig 3, 4, S3, S4, S5
4	TTCTCCGCACCATTCG	Used in Fig 3, 4, S3, S4, S5
5	TCTGGACACACCGTAA	Used in Fig 3, 4, S3, S4, S5
6	TAGCATTGTCACCTTC	Used in Fig 3, 4, S3, S4, S5

Table S2: List of DNA barcodes used in this study
